# Postprocedural Anticoagulation After Primary Percutaneous Coronary Intervention: 1-Year Results From the RIGHT Trial and Meta-analyses

**DOI:** 10.1016/j.jacasi.2026.04.003

**Published:** 2026-05-22

**Authors:** Yan Yan, Xiao Wang, Zeyuan Fan, Jincheng Guo, Guozhong Wang, Delu Yin, Zhifang Wang, Fuchun Zhang, Changming Tian, Wei Gong, Jiamin Liu, Jiapeng Lu, Changsheng Ma, Eric Vicaut, Gilles Montalescot, Shaoping Nie, RIGHT Investigators

**Affiliations:** a Center for Coronary Artery Disease, Division of Cardiology, Beijing Anzhen Hospital, Capital Medical University, Beijing, China; b National Clinical Research Center of Cardiovascular Diseases, Beijing, China; c Beijing Institute of Heart, Lung, and Blood Vessel Diseases, Beijing, China; d Cardiometabolic Medicine Center, Fuwai Hospital, National Center for Cardiovascular Diseases, Chinese Academy of Medical Sciences and Peking Union Medical College, Beijing, China; e Civil Aviation General Hospital, Beijing, China; f Beijing Luhe Hospital, Capital Medical University, Beijing, China; g The First People's Hospital of Lianyungang, Jiangsu, China; h Xinxiang Central Hospital, Henan, China; i Beijing Haidian Hospital, Beijing, China; j The People's Hospital of Yongcheng, Henan, China; k National Clinical Research Center for Cardiovascular Diseases, NHC Key Laboratory of Clinical Research for Cardiovascular Medications, State Key Laboratory of Cardiovascular Disease, Fuwai Hospital, Chinese Academy of Medical Sciences and Peking Union Medical College, National Center for Cardiovascular Diseases, Beijing, China; l National Clinical Research Center of Cardiovascular Diseases, State Key Laboratory of Cardiovascular Disease, Fuwai Hospital, National Center for Cardiovascular Diseases, Chinese Academy of Medical Sciences and Peking Union Medical College, Beijing, China; m Arrhythmia Center, Division of Cardiology, Beijing Anzhen Hospital, Capital Medical University, Beijing, China; n ACTION Study Group, Epidemiology and Clinic Research Unit, Lariboisière University Hospital, Paris, France; o ACTION Study Group, Sorbonne Université, INSERM UMRS 1166, Institut de Cardiologie, Hôpital Pitié-Salpêtrière (AP-HP), Paris, France

**Keywords:** follow-up, postprocedural anticoagulation, primary percutaneous coronary intervention, ST-segment elevation myocardial infarction

## Abstract

**Background:**

The RIGHT trial was designed to assess the efficacy and safety of postprocedural anticoagulation (PPA) in patients with ST-segment elevation myocardial infarction undergoing primary percutaneous coronary intervention (PCI).

**Objectives:**

The authors aimed to report the prespecified 1-year outcomes.

**Methods:**

RIGHT is an investigator-initiated, multicenter, randomized, double-blind, placebo-controlled, superiority trial conducted in 53 sites across China. Patients with ST-segment elevation myocardial infarction were randomly assigned (1:1) after primary PCI to receive low-dose PPA (enoxaparin, unfractionated heparin, or bivalirudin) or matching placebo for at least 48 hours. Major adverse cardiac events (MACEs) including all-cause death, nonfatal myocardial infarction, nonfatal stroke, stent thrombosis (definite), and urgent revascularization (any vessel), were assessed during a 1-year follow-up.

**Results:**

Over a median follow-up of 1.0 year (IQR: 1.0-1.0), MACE data were available for 99.2% of participants. MACEs occurred in 4.2% (63/1,494) of the PPA group and 4.9% (73/1,495) of the placebo group (HR: 0.86; 95% CI: 0.61-1.21), with no between-group difference in major bleeding (1.3% vs 1.5%; HR: 0.87; 95% CI: 0.47-1.62). In the group of enoxaparin vs placebo, we observed reduction of MACEs with enoxaparin (HR: 0.53; 95% CI: 0.30-0.97) with no excess bleeding. Meta-analyses also showed an advantage of enoxaparin over no anticoagulation in reducing MACEs at 30 days (risk ratio: 0.635; 95% CI: 0.399-0.997).

**Conclusions:**

Low-dose PPA after primary PCI was safe but did not reduce ischemic events at the 1-year follow-up. If clinically indicated, our results suggest that enoxaparin may be beneficial and warrants confirmation in future studies. (Comparison of Anticoagulation Prolongation vs. no Anticoagulation in STEMI Patients After Primary PCI [RIGHT]; NCT03664180)

## Central Illustration

**Figure:**
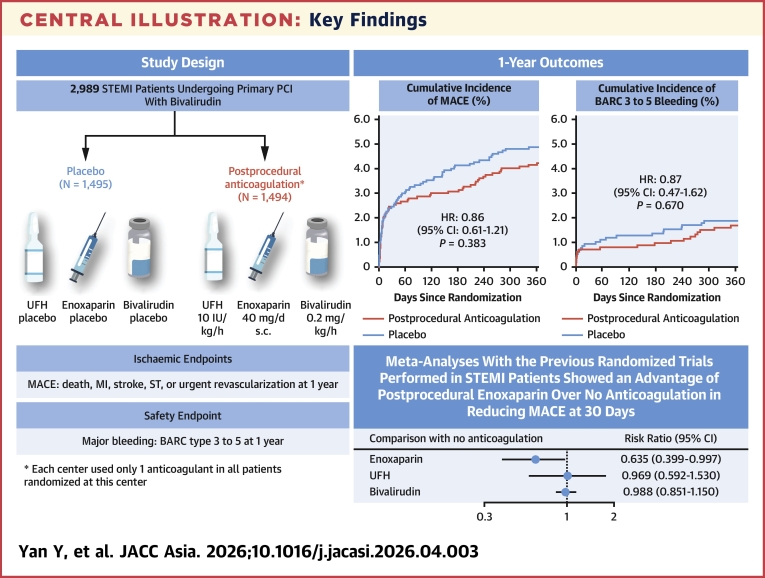

Empirical prescription of postprocedural anticoagulation (PPA) after primary percutaneous coronary intervention (PCI) is frequent, aiming to prevent persistent thrombin generation after ST-segment elevation myocardial infarction (STEMI).^1^ However, data regarding the efficacy, safety, and optimal regimens of routine PPA are scarce, especially when it comes to long-term follow-up. The current European and U.S. guidelines do not provide recommendations for PPA in STEMI patients.^2^^,^^3^

Results of post-hoc studies in a large patient population undergoing primary PCI by means of full-dose or low-dose postprocedural bivalirudin in comparison with unfractionated heparin (UFH) have been cited by the clinical and scientific communities to provide objective evidence in STEMI. Thus, we performed a multicenter, randomized trial, the RIGHT (Randomized comparison of anticoagulation after primary percutaneous coronary Intervention using enoxaparin, ACT Guided unfractionated Heparin or bivalirudin prolongation vs no anticoagulation To improve clinical outcome) trial, to compare PPA and placebo after a full-dose bivalirudin infusion after primary PCI. Overall, 30-day results showed no substantial clinical differences between routine PPA and placebo in this low-to-moderate risk population, although heterogeneity was observed across the 3 anticoagulants used.^4^

In fact, only 6 randomized trials administering PPA after primary PCI have been conducted, with different anticoagulants and discordant results.^4^, ^5^, ^6^, ^7^, ^8^, ^9^, ^10^, ^11^ We undertook the RIGHT trial to compare the efficacy and safety of PPA and each anticoagulant in STEMI patients. We hypothesized that PPA with specific anticoagulants was superior to no anticoagulation in reducing the incidence of major adverse cardiovascular events (MACEs).

## Methods

### Study design and patients

The RIGHT trial was an investigator-initiated, multicenter, randomized, double-blind, placebo-controlled, superiority trial in STEMI patients performed in 53 centers across China. The study design, eligibility criteria, endpoints, and statistical analysis were described previously, and patient outcomes to 30 days have been reported.^4^^,^^12^ Patients eligible for randomization were those who had STEMI and underwent primary PCI within 12 hours, excluding those in shock and those who received lytic therapy before catheterization. Enrolled patients were randomized at 1:1 to receive low-dose PPA or matching placebo for at least 48 hours. Before trial initiation, each center selected 1 from the following 3 PPA regimens: enoxaparin 40 mg once daily subcutaneously, UFH 10 units/kg/h intravenously adjusted to maintain activated clotting time between 150 and 220 seconds, and bivalirudin 0.2 mg/kg/h intravenously. Study assessments were performed at baseline, at 48 hours or discharge (whichever was earlier), and at follow-ups of 30 days, 6 months, and 1 year.

All patients or their legal representatives provided written informed consent. All participating centers obtained ethics approval from institutional review boards. Study oversight consisted of a steering committee that provided scientific direction, a clinical events committee that adjudicated selected safety and efficacy endpoints while blinded to the assigned study drug, and an independent data safety monitoring board that reviewed the endpoints and serious adverse events throughout the trial. The RIGHT trial was sponsored by Beijing Hospitals Authority Clinical Medicine Development of special funding support and Jiangsu Hengrui Pharmaceuticals provided funding for the trial through a research grant to the Beijing United Heart Foundation. The ACTION study group provided methodological, statistical, and operational support for the conduct of the trial as well as editorial assistance for the manuscript. The RIGHT trial was registered (NCT03664180) and has been completed.

### Study endpoints

The primary outcomes were collected at the 30-day follow-up, including the efficacy endpoint of MACEs and the safety endpoint of Bleeding Academic Research Consortium (BARC) types 3 to 5 major bleeding.^13^ MACEs were defined as a composite of all-cause death, nonfatal myocardial infarction, nonfatal stroke, stent thrombosis (definite), or urgent revascularization (any vessel). All primary outcomes, as well as individual events, were re-evaluated at the 1-year follow-up with the same procedure and checked by the Chinese Center for Disease Control and Prevention's national death surveillance system.^14^ This study presents the prespecified final 1-year outcomes of the entire project.

### Statistical analysis

Primary analyses of the efficacy and safety endpoints were performed in the intention-to-treat and safety populations, respectively. The cumulative incidences of the primary efficacy and safety endpoints were estimated using the Kaplan-Meier method and compared with the log-rank test. HRs and 95% CIs were estimated using Cox proportional hazards models, with adjustments made for study center as a random effect. HRs and 95% CIs for the secondary ischemic and safety endpoints were estimated using mixed-effects Cox proportional hazard regression models as described above. The Hartung-Knapp-Sidik-Jonkman estimator was used to estimate between-study variance in our meta-analysis. The proportional hazards assumption for the Cox models was assessed using the Schoenfeld residual test, and no violation was found (Supplemental Table 1). We assessed the secondary endpoints according to a hierarchical procedure to control for multiple comparisons. In this procedure, *P* value was reported only until the last comparison for which the *P* value was significant. Further details can be found in the Statistical Analysis Plan.^4^ Missing data at baseline were not imputed. Missing data for the primary endpoint were censored at the time of the last available information.

Sensitivity analyses were conducted for all study endpoints in the per-protocol population. In addition, sensitivity analyses on the primary efficacy and safety endpoints were performed using Cox models adjusted for age, sex, body mass index, diabetes, hypertension, peripheral artery disease, previous stroke, and study center as a random effect. We also performed stratified analyses in the following prespecified subgroups using multiplicative interactions terms: age (>75 vs ≤75 years), sex, history of diabetes mellitus (yes vs no), prior cancer (yes vs no), prior stroke (yes vs no), prior PCI (yes vs no), body weight (<60 kg vs ≥60 kg), creatinine clearance (<30 mL/min vs ≥30 mL/min), location of myocardial infarction (anterior vs nonanterior), anticoagulant before angiography (yes vs no), glycoprotein IIb/IIIa inhibitor bailout use (yes vs no), type of stent (drug-eluting stent vs bare-metal stent), and total length of stent (>60 mm vs ≤60 mm). All analyses were 2-sided at a 5% significance level and performed using SAS version 9.4 (SAS Institute).

### Meta-analyses

Additionally, we conducted a quantitative meta-analysis by combining our results with those of previous STEMI trials performed in patients undergoing primary PCI with PPA by calculating the risk ratio (RR) and 95% CIs for each trial (Supplemental Table 2). The PubMed, MEDLINE, and EMBASE international databases were systematically searched for STEMI studies that reported PPA from their inception until June 2024. Two authors (Y.Z. and H.T.) independently conducted the search at title and abstract levels. Studies potentially eligible were independently reviewed by these authors at the full-text level and subsequently included. Any disagreements were resolved by a third senior author (Y.Y.).

The primary inclusion criterion was the availability of sufficient raw data to calculate study-specific RRs for 30-day MACEs (Supplemental Methods). Only studies involving patients undergoing primary PCI were included. The quality of the included studies was independently assessed by 2 reviewers using the Cochrane Risk of Bias tool.^15^ Two authors independently extracted data from each study by reviewing the published articles and supplementary materials (Supplemental Methods, Supplemental Figure 1). Statistical heterogeneity was summarized using the I^2^ statistic test. An I^2^ value <25% was considered to indicate low heterogeneity, a value between 25% and 50% was considered moderate heterogeneity, and a value >50% was considered substantial heterogeneity, according to the Cochrane guidelines. If between-study heterogeneity was not substantial, the fixed-effect model with the Mantel-Haenszel method was used to calculate the summarized RR; otherwise, the random effects model was used. Inverse-variance weighting was used in the meta-analysis. Publication bias was evaluated by a funnel plot, Begg’s test, and Egger’s test.

To further compare the effectiveness of each specific regimen with that of placebo, a network meta-analysis was conducted. First, network geometry was used to explore the comparative relationships among interventions. Then, a Bayesian network meta-analysis model was used to synthesize the study effect sizes. RRs with 95% CIs for MACEs were presented for each intervention comparison. Consistency evaluation was conducted by node-splitting analysis. Finally, intervention ranking was assessed using the surface under the cumulative ranking curve plot, where values closer to 1 indicate superior efficacy.

This systematic review and network meta-analysis followed the Preferred Reporting Items for Systematic Reviews and Meta-Analyses guidelines for network meta-analyses.^16^ The *P* values were 2-tailed, reaching a statistically significant level at 0.05. The meta-analysis and Bayesian network meta-analyses were conducted using R 4.4.1 (R Foundation for Statistical Computing).

## Results

### Baseline characteristics

Between January 11, 2019 and September 18, 2021, 2,989 participants were enrolled and assigned to either the PPA group (n = 1,494) or the placebo group (n = 1,495). Baseline demographics and procedural characteristics were similar between the 2 groups (Supplemental Table 3). The median duration of the study medication administration was consistent across both the PPA and placebo groups (48.0 hours [IQR: 48.0-56.2] and 48.0 hours [IQR: 48.0-57.1], respectively) and was similar among the different anticoagulants (enoxaparin, UFH, or bivalirudin; 48.1 hours [IQR: 48.0-72.0], 48.0 hours [IQR: 48.0-48.9], or 48.0 hours [IQR: 48.0-50.0], respectively). Data for the primary outcome were available for 2,964 (99.2%) of the 2,989 participants at the 1-year follow-up (completed in November 2022) compared with 2,980 (99.7%) of the 2,989 participants at the 30-day follow-up (Supplemental Figure 2).

### 1-year clinical outcomes

**Figure 1:**
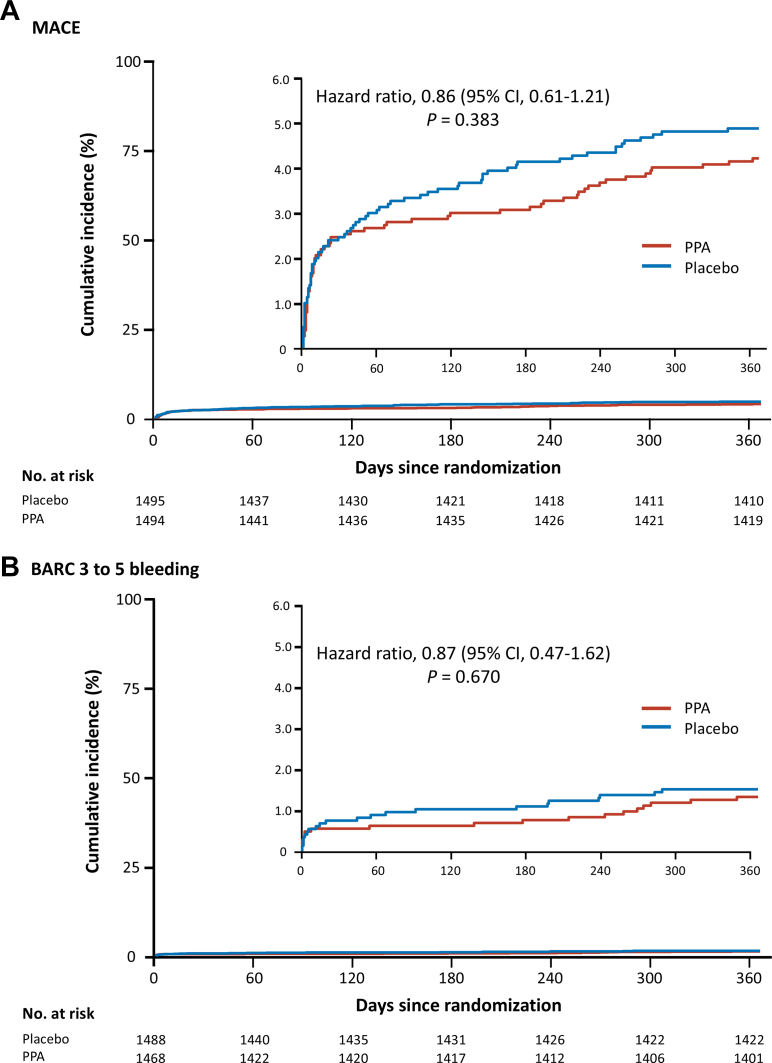
1-Year Clinical Outcomes in PPA vs Placebo The primary objective was PPA vs placebo. (A) MACEs included all-cause death, nonfatal myocardial infarction, nonfatal stroke, stent thrombosis (definite). or urgent revascularization (any vessel) over 1-year of follow-up. (B) BARC types 3 to 5 bleeding through 1 year from randomization. BARC = Bleeding Academic Research Consortium; MACE = major adverse cardiovascular event; PPA = postprocedural anticoagulation.

Over a median follow-up of 1 year (IQR: 1.0-1.0), MACEs occurred in 4.2% (63/1,494) of patients treated with PPA compared with 4.9% (73/1,495) of patients treated with placebo (HR: 0.86; 95% CI: 0.61-1.21) (Table 1, Figure 1A). The incidence of BARC type 3 to 5 bleeding did not differ significantly between patients who received PPA and those who did not (1.3% [19/1,468] vs 1.5% [22/1,488]; HR: 0.87; 95% CI: 0.47-1.62) (Table 1, Figure 1B). Event rates for other endpoints are shown in Table 1. As shown in Supplemental Figures 3A and 3B, the effects of PPA vs placebo on the efficacy and safety endpoints were largely consistent across the prespecified subgroups.

**Table 1:** Clinical Outcomes in the Global Population at 1 Year^a^

	Postprocedural Anticoagulation		Placebo		HR (95% CI)	*P* Value
	No. of Patients With Event	Outcome Rates and 95% CIs Derived From Kaplan-Meier Analysis (%)	No. of Patients With Event	Outcome Rates and 95% CI Derived From Kaplan-Meier Analysis (%)		
Efficacy endpoints	(n = 1,494)		(n = 1,495)			
Major adverse cardiovascular events^b^	63	4.2 (3.1-5.2)	73	4.9 (3.6-5.8)	0.86 (0.61-1.21)	0.383
All-cause death, nonfatal MI, nonfatal stroke, or urgent revascularization	63	4.2 (3.1-5.2)	73	4.9 (3.6-5.8)	0.86 (0.61-1.21)	
All-cause death, nonfatal MI, or nonfatal stroke	61	4.1 (3.1-5.2)	72	4.8 (3.5-5.7)	0.84 (0.6-1.19)	
Cardiovascular death or nonfatal MI	48	3.2 (2.0-3.8)	44	2.9 (2.4-4.3)	1.21 (0.76-1.90)	
Definite stent thrombosis (Academic Research Consortium definition)	4	0.3 (0.0-0.5)	4	0.3 (0.0-0.6)	1.00 (0.25-4.00)	
Cardiovascular death	41	2.7 (2.0-3.8)	34	2.3 (1.4-2.9)	1.21 (0.77-1.90)	
All-cause death	43	2.9 (2.1-3.9)	37	2.5 (1.5-3.1)	1.16 (0.75-1.81)	
Nonfatal MI	17	1.1 (0.5-1.6)	26	1.7 (1.1-2.5)	0.65 (0.35-1.20)	
Nonfatal stroke	10	0.7 (0.2-1.0)	11	0.7 (0.2-1.1)	0.91 (0.39-2.14)	
Safety endpoints	(n = 1,468)		(n = 1,488)			
BARC types 3-5 bleeding	19	1.3 (0.7-1.9)	22	1.5 (0.9-2.1)	0.87 (0.47-1.62)	0.67
BARC types 1, 2, 3, 4, and 5	215	14.6 (13.0-16.6)	217	14.6 (12.9-16.6)	1.01 (0.84-1.22)	
BARC types 2, 3, or 5	59	4.0 (3.0-5.1)	58	3.9 (2.9-4.9)	1.04 (0.72-1.49)	
TIMI flow grade major, minor, minimal bleeding, and their combination	60	4.1 (3.1-5.1)	58	3.9 (2.9-4.9)	1.05 (0.73-1.51)	
STEEPLE major bleeding	18	1.2 (0.7-1.8)	22	1.5 (0.9-2.1)	0.83 (0.44-1.54)	
GUSTO severe or moderate bleeding	12	0.8 (0.4-1.3)	12	0.8 (0.4-1.3)	1.01 (0.45-2.25)	
Thrombocytopenia	4	0.3 (0.0-0.5)	0	0.0 (0.0-0.0)	—	

BARC = Bleeding Academic Research Consortium; GUSTO = Global Utilization of Streptokinase and Tissue Plasminogen Activator for Occluded Coronary Arteries; MI = myocardial infarction; STEEPLE = Safety and Efficacy of Enoxaparin in Percutaneous Coronary Intervention Patients.

a The analysis of the efficacy indicators was carried out in an intention-to-treat set, and analysis of the safety endpoints were carried out in the safety set.

b Major adverse cardiovascular events were all-cause death, nonfatal MI, nonfatal stroke, stent thrombosis (definite), or urgent revascularization (any vessel).

**Figure 2:**
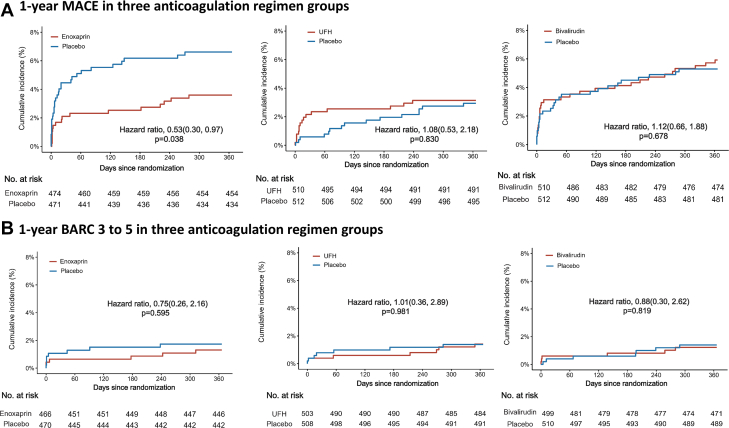
1-Year Clinical Outcomes in 3 Anticoagulation Regimens (A) 1-year MACEs among patients treated with enoxaparin, UFH, or bivalirudin. (B) 1-year BARC types 3 to 5 bleeding among patients treated with enoxaparin, UFH, or bivalirudin. UFH = unfractionated heparin; Abbreviations as in Figure 1.

**Figure 3:**
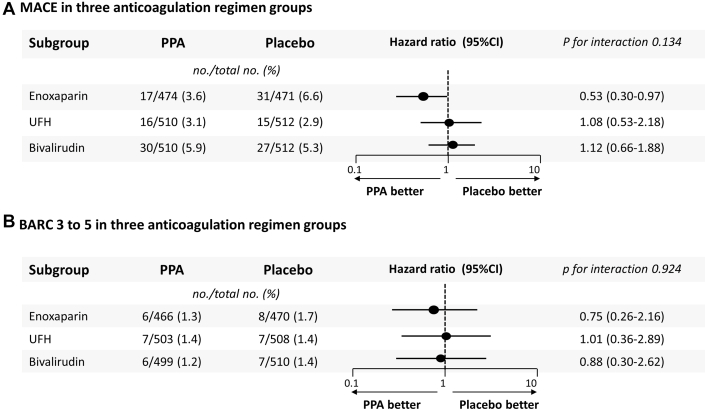
Efficacy and Safety Outcomes in 3 Anticoagulation Regimens vs Placebo The key secondary objective was the effect of each anticoagulation regimen (enoxaparin, UFH, or bivalirudin) vs placebo. (A) 1-year MACE. (B) 1-year BARC types 3 to 5 bleeding. Abbreviations as in Figures 1 and 2.

There was apparent heterogeneity in the effect of the 3 anticoagulants over the 1-year follow-up (Figure 2, Supplemental Table 4). The incidence of the primary efficacy endpoint was lower with enoxaparin than placebo (3.6% [17/474] vs 6.6% [31/471]; HR: 0.53; 95% CI: 0.30-0.97), whereas no significant difference was observed with bivalirudin vs placebo (HR: 1.12; 95% CI: 0.66-1.88) and UFH vs placebo (HR: 1.08; 95% CI: 0.53-2.18) (Figure 3).

### Meta-analysis

A total of 791 studies were initially identified through comprehensive searches across multiple databases. After removing duplicated studies, 573 studies remained. After title and abstract screening, 162 articles were excluded. After full-text assessment, an additional 155 studies were removed based on the eligibility criteria. Finally, 7 studies were included in the analysis (Supplemental Figure 4). Differences in baseline characteristics, designs, and results are shown in Supplemental Table 2. Funnel plot, Begg’s test (*P* = 0.55), and Egger’s test (*P* = 0.87) indicated no significant publication bias among all included studies (Supplemental Figure 5). The I^2^ was 50.6% (range, 0.0%-79.0%) and τ^2^ was 0.034 (range, 0.000-0.256), which indicated significant between-study heterogeneity (Supplemental Results).

**Figure 4:**
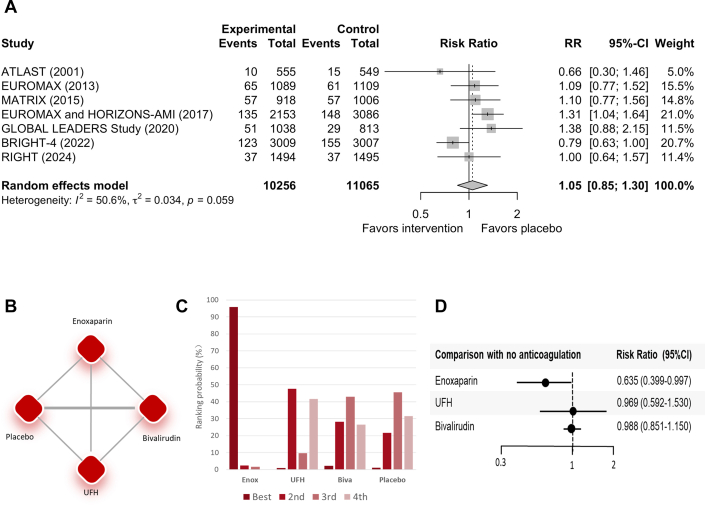
Meta-Analyses Meta-analysis estimates of PPA effectiveness in MACEs during the 30-day period compared with no PPA (A) or network analysis of 3 anticoagulation treatments (enoxaparin, UFH, or bivalirudin) vs placebo with network plot (B), ranking plot (C), and forest plot (D). A regimen that includes enoxaparin seems to be the most favorable treatment option and may be the preferred PPA for most patients with ST-segment elevation myocardial infarction after primary percutaneous coronary intervention. Abbreviations as in Figures 1 and 2.

The overall risk of MACEs at 30 days was not significantly different between patients with PPA and those without (RR: 1.05; 95% CI: 0.89-1.25), as shown in Figure 4A. There was significant heterogeneity across the 3 anticoagulants in terms of MACEs at 30 days (Figures 3, and 4B to 4D). No significant inconsistency was identified based on node-splitting analysis (*P* = 0.90). Postprocedural enoxaparin was associated with a lower risk (RR: 0.635; 95% CI: 0.399-0.997), whereas UFH and bivalirudin exhibited RRs of 0.969 (95% CI: 0.592-1.53) and 0.988 (95% CI: 0.851-1.15), respectively.

## Discussion

We conducted a randomized trial comparing low-dose PPA (using 3 different anticoagulants) with no anticoagulation (matching placebos) in low-to-moderate risk patients with STEMI (Central Illustration). Although low-dose PPA administered for at least 48 hours after primary PCI was safe, it did not prove to be superior to no anticoagulation in reducing ischemic outcomes at 1 year. However, low-dose enoxaparin improved the risk-to-benefit ratio compared with placebo at 1 year in contrast to the other 2 anticoagulants. Our results in the RIGHT trial seem to be confirmed by the meta-analyses of 6 randomized trials.

**Central Illustration:**
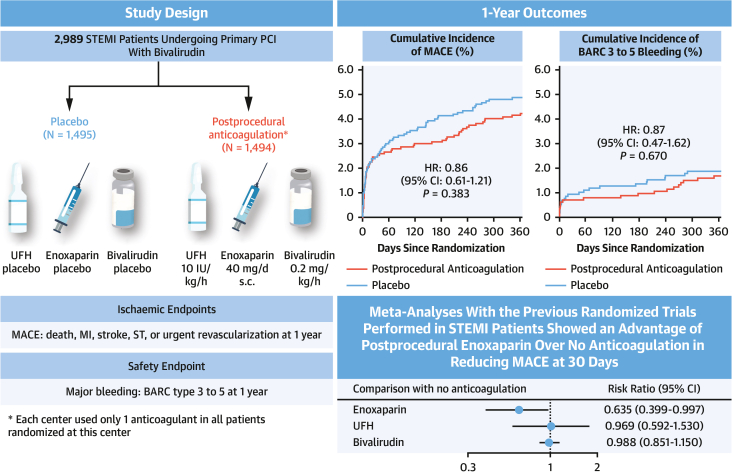
Key Findings Study design and 1-year outcomes of the RIGHT trial. A total of 2,989 patients with STEMI undergoing primary PCI with bivalirudin were randomized to placebo or low-dose PPA with UFH, enoxaparin, or bivalirudin, according to center assignment. Kaplan-Meier curves show no significant difference between groups in MACEs or BARC type 3 to 5 bleeding at 1 year. The meta-analyses from prior randomized trials suggests a reduction in 30-day MACEs with enoxaparin vs no anticoagulation. PCI = percutaneous coronary intervention; STEMI = ST-segment elevation myocardial infarction; Abbreviations as in Figures 1 and 2.

Our 1-year analysis, with no time-dependent divergence, confirms the 30-day findings indicating that low-dose PPA provides no benefit in reducing ischemic events and shows no sign of harm in patients with STEMI. In contrast to previous studies, participants in our trial were eligible for randomization only if they received intravenous bivalirudin during and immediately after primary PCI, with a full dose of 1.75 mg/h required before PPA initiation.^8^, ^9^, ^10^, ^11^^,^^17^ Despite consistent enrollment criteria with previous trials, the baseline characteristics may reflect a selection bias, as centers appeared to recruit patients at relatively low risk after successful procedures, potentially attenuating the benefit of PPA. In high-risk STEMI presentations (such as cardiogenic shock, angiographic no-reflow, or left ventricular thrombus), PPA has been reported in selected cohorts and may be associated with potential clinical benefit.^18^^,^^19^ Future studies with comprehensive procedural and angiographic data collection will be required to definitively evaluate treatment effects within the complex high-risk PCI population.

Extended follow-up up to 1 year confirmed the findings observed at 30 days, showing that PPA was not superior to placebo. However, the 3 anticoagulants behaved differently in preventing ischemic events over 1 year of follow-up, confirming the 30-day data. Enoxaparin demonstrated a lower incidence of MACEs at 30 days, with a consistent reduction at 1 year. These findings are consistent with those of previous studies but should be interpreted with caution. Although age and prior myocardial infarction were assessed for transitivity, key effect modifiers, such as the number of premature termination of investigational products and duration of investigational products, were poorly reported in most studies, which limited the reliability of indirect comparisons (Supplemental Table 4). Although prespecified, the comparison between anticoagulants remains hypothesis-generating given the study design and lack of power for interaction analysis.^20^, ^21^, ^22^ Nevertheless, in centers allocated to enoxaparin, the randomized double-blind comparison favored enoxaparin PPA over placebo, a result not observed in centers allocated to UFH or bivalirudin PPA.

A biologically plausible explanation for the observed separation with enoxaparin is that in the hours to days after PCI, when tissue factor–driven coagulation remains heightened and dual-antiplatelet therapy primarily targets platelet activation, low–molecular-weight heparin may provide a steadier “bridging” inhibition of coagulation through its more predictable anticoagulation compared with UFH and broader inhibition (anti-factor Xa and anti-IIa) compared with bivalirudin. In addition, this is obtained without infusion and with lower interpatient variability (less nonspecific protein/cell binding) and without anticoagulation monitoring. These results are aligned with similar superiority of enoxaparin reported in the past.^6^^,^^9^, ^10^, ^11^^,^^22^ Given the significance of this question, we updated the previous literature search and network meta-analysis to provide a state-of-the-art evidence base on different types of PPA in the STEMI population. In this updated network meta-analysis, a parenteral anticoagulant regimen that includes prophylactic use of enoxaparin after primary PCI appears to be associated with fewer ischemic complications while maintaining safety. Although heterogeneity sources provided substantial clinical differences across trials (eg, glycoprotein IIb/IIIa inhibitor use: 3.3%-77%; radial access: 23.8%-98.6%), our data suggest that a regimen of 40 mg enoxaparin once daily has the best safety profile (number needed to treat for MACEs: 33.3) and may represent a promising strategy that warrants evaluation in adequately powered, confirmatory randomized trials in the future.

Most guideline recommendations for PPA are based on low-quality evidence, often relying on extrapolation from observational studies. Remarkable advances have been made in recent years, starting with the initial findings from the MATRIX (Minimizing Adverse Hemorrhagic Events by Transradial Access Site and Systemic Implementation of Angiox) trial in patients with acute coronary syndrome, followed by the RIGHT trial in STEMI.^4^^,^^17^ Together, these studies provide high-quality data from more than 21,000 patients, offering meaningful insights. Given these findings and in line with the latest European Society of Cardiology guidelines, PPA is broadly not recommended for routine use after invasive procedures, especially in patients at low risk or recurrent events.^2^

### Study limitations

The design of the RIGHT trial resulted in a selective population with a low event rate, which may limit the generalizability of the findings to broader populations. The trial population was restricted to patients enrolled in China; therefore, the generalizability of these findings to other geographic regions and ethnic populations remains uncertain. Additionally, the 3 anticoagulants were not directly comparable because of the absence of central randomization between the 3 anticoagulants (for practical reasons), but all patients were randomized between 1 anticoagulant and the matching placebo, and the trial was not powered for interaction analysis. The double-blind nature of the study and the blinded event adjudication by the clinical event committee, however, ensures the absence of bias in the reported events. Considering the inherent difficulties of a central randomization for the type of drug (emergent situation, blinded pharmaceutical circuit for 3 drugs and 3 placebos at each center, cost issues, etc), the type of anticoagulant was not centrally allocated, but local randomization and blinding occurred for each patient for the drug selected by the center. We cannot exclude inherent center-level confounding with this design. Although the use of network meta-analysis enables simultaneous comparisons and evidence-based grading to facilitate overall conclusions, we believe it lacks the granularity to address specific anticoagulants. Another limitation is the lower than expected event rate, as frequently encountered in trials of recent acute myocardial infarction. This may have reduced statistical power and contributed to the wide CI around the HR. Based on our observed effect estimate (HR: 0.53) and 1-year MACE rates (3.6% with enoxaparin vs 6.6% with placebo), a confirmatory trial would require approximately 859 patients per group (≈1,718 total) to ensure adequate power.

## Conclusions

Our results complement previous work suggesting that routine PPA after primary PCI is safe but does not improve ischemic outcomes. The data are consistent throughout the trial from 30 days to 1 year and in the network meta-analysis. Moreover, in cases where PPA is clinically warranted, our results consistently indicate that enoxaparin effectively reduces the risk of MACEs after primary PCI.

## Funding Support and Author Disclosures

The RIGHT study was supported by Beijing Hospitals Authority Clinical Medicine Development of special funding support (ZLRK202318) and Jiangsu Hengrui Pharmaceuticals through a research grant to the Beijing United Heart Foundation (BJUHFRIGHT201802). Dr Yan received grants from the National Natural Science Foundation of China (82470259, 82100260), Beijing Natural Science Foundation (L2510002) and has received consulting fees from Idorsia and Viatris. Dr X. Wang has received grants from National Key Research & Development Program of China (2022YFC2505600), Beijing Municipal Natural Science Foundation Grant (JQ24039), National Natural Science Foundation of China (82470339), and Outstanding Young Talent of National High-Level Personnel of Special Support Program (2024-RWS01). Dr Ma has received speaker fees from Bristol-Myers Squibb, Pfizer, Johnson & Johnson, Boehringer-Ingelheim, Bayer, and AstraZeneca. Dr Vicaut has received consulting fees from Abbott and Bristol Myers Squibb. Dr Montalescot has received research grants to his institution or consulting/lecture fees from Abbott, Amgen, AstraZeneca, Ascendia, Bayer, Bristol-Myers Squibb, Boehringer-Ingelheim, Boston Scientific, Celecor, CSL Behring, Idorsia, Lilly, Novartis, Novo, Opalia, Pfizer, Quantum Genomics, Sanofi, and Terumo. Dr Nie was funded by Beijing Hospitals Authority Clinical Medicine Development of special funding support (ZLRK202318), National Natural Science Foundation of China (82270258), and Beijing Municipal Science & Technology Commission, China (Z221100003522027) and has received research grants to his institution from Boston Scientific, Abbott, Jiangsu Hengrui Pharmaceuticals, China Resources Sanjiu Medical & Pharmaceuticals, and East China Pharmaceuticals. All other authors have reported that they have no relationships relevant to the contents of this paper to disclose.
