# Supplementary material for: Postprocedural Anticoagulation After Primary Percutaneous Coronary Intervention: 1-Year Results From the RIGHT Trial and Meta-analyses
Source: JACC Asia. 2026 May 22;6(7):1095–105. doi: 10.1016/j.jacasi.2026.04.003 (PMC13350653; doi:10.1016/j.jacasi.2026.04.003)
Supplement: Supplemental Material [file mmc1.docx]

**Postprocedural Anticoagulation after Primary PCI: 1-Year Results from the RIGHT Trial and** **Meta-analyses**

Short title: 1-Year Outcomes with PPA after Primary PCI

Yan Yan, M.D. ^a,b,c^, Xiao Wang, M.D. ^a,d^, Zeyuan Fan, M.D. ^e^, Jincheng Guo, M.D.^f^, Guozhong Wang, M.D.^f^, Delu Yin, M.D. ^g^, Zhifang Wang, M.D. ^h^, Fuchun Zhang, M.D. ^i^, Changming Tian, M.D. ^j^, Wei Gong, M.D. ^a,b,c^, Jiamin Liu, M.D. ^k^, Jiapeng Lu, Ph.D. ^l^, Changsheng Ma, M.D., Ph.D. ^b,c,m^, Eric Vicaut, M.D., Ph.D. ^n^, Gilles Montalescot, M.D., Ph.D. ^o^ and Shaoping Nie, M.D., Ph.D. ^a,b,c^, on behalf of the [RIGHT Investigators](https://pubmed.ncbi.nlm.nih.gov/?term=CCC-ACS+Investigators%5BCorporate+Author%5D)

^a^ Center for Coronary Artery Disease, Division of Cardiology, Beijing Anzhen Hospital, Capital Medical University, Beijing, China

^b^ National Clinical Research Center of Cardiovascular Diseases, Beijing, China

^c^ Beijing Institute of Heart, Lung, and Blood Vessel Diseases, Beijing, China

^d^ Cardiometabolic Medicine Center, Fuwai Hospital, National Center for Cardiovascular Diseases, Chinese Academy of Medical Sciences and Peking Union Medical College, Beijing, China

^e^ Civil Aviation General Hospital, Beijing, China

^f^ Beijing Luhe Hospital, Capital Medical University, Beijing, China

^g^ The First People's Hospital of Lianyungang, Jiangsu, China

^h^ Xinxiang Central Hospital, Henan, China

^i^ Beijing Haidian Hospital, Beijing, China

^j^ The People's Hospital of Yongcheng, Henan, China

^k^ National Clinical Research Center for Cardiovascular Diseases, NHC Key Laboratory of Clinical Research for Cardiovascular Medications, State Key Laboratory of Cardiovascular Disease, Fuwai Hospital, Chinese Academy of Medical Sciences and Peking Union Medical College, National Center for Cardiovascular Diseases, Beijing, China

^l^ National Clinical Research Center of Cardiovascular Diseases, State Key Laboratory of Cardiovascular Disease, Fuwai Hospital, National Center for Cardiovascular Diseases, Chinese Academy of Medical Sciences and Peking Union Medical College, Beijing, China

^m^ Arrhythmia center, Division of Cardiology, Beijing Anzhen Hospital, Capital Medical University, Beijing, China

^n^ ACTION Study Group, Epidemiology and Clinic Research Unit, Lariboisière University Hospital, Paris, France

^o^ ACTION Study Group, Sorbonne Université, INSERM UMRS 1166, Institut de Cardiologie, Hôpital Pitié-Salpêtrière (AP-HP), Paris, France

**ADDRESS FOR CORRESPONDENCE**:

Gilles Montalescot, MD, PhD

ACTION Study Group, Institut de Cardiologie,

Hôpital Pitié-Salpêtrière, Paris, France, 75013

E-mail: gilles.montalescot@aphp.fr

Shaoping Nie, MD, PhD

Emergency & Critical Care Center

Beijing Anzhen Hospital, Capital Medical University

2 Anzhen Road, Chaoyang District

Beijing, China, 100029

Email: spnie@ccmu.edu.cn

Fax: +86 10 8400 5254

Twitter handle: @ActionCoeur

# TWEET

In STEMI patients, low-dose anticoagulants post-primary PCI were safe but didn’t reduce 1-year ischemic events. (RIGHT trial; NCT03664180)

**SOURCES OF FUNDING**

The RIGHT study was supported by Beijing Hospitals Authority Clinical Medicine Development of special funding support (ZLRK202318) and Jiangsu Hengrui Pharmaceuticals through a research grant to the Beijing United Heart Foundation (BJUHFRIGHT201802).

**DISCLOSURES**

Dr Yan was funded by grants from the National Natural Science Foundation of China (82470259, 82100260) and reports consulting fees from Idorsia and Viatris. Dr X. Wang was funded by grants from National Key Research & Development Program of China (2022YFC2505600), Beijing Municipal Natural Science Foundation Grant (JQ24039), National Natural Science Foundation of China (82470339), and Outstanding Young Talent of National High-Level Personnel of Special Support Program (2024-RWS01). Dr Ma reports honoraria from Bristol-Myers Squibb (BMS), Pfizer, Johnson & Johnson, Boehringer-Ingelheim (BI), Bayer and AstraZeneca for giving lectures. Dr Vicaut reports consulting fees from Abbott and Bristol Myers Squibb. Dr Montalescot reports research grants to the Institution or Consulting/Lecture Fees from Abbott, Amgen, AstraZeneca, Ascendia, Bayer, BMS, Boehringer-Ingelheim, Boston-Scientific, Celecor, CSL Behring, Idorsia, Lilly, Novartis, Novo, Opalia, Pfizer, Quantum Genomics, Sanofi, Terumo. Dr Nie was funded by Beijing Hospitals Authority Clinical Medicine Development of special funding support (ZLRK202318), National Natural Science Foundation of China (82270258), Beijing Municipal Science & Technology Commission, China (Z221100003522027) and reports research grants to the Institution from Boston Scientific, Abbott, Jiangsu Hengrui Pharmaceuticals, China Resources Sanjiu Medical & Pharmaceuticals, East China Pharmaceuticals. The other authors have no conflicts of interest to declare.

**ACKNOWLEDGMENTS**

The authors thank associate researcher Chaoqun Wu and assistant professor Xueke Bai (National Clinical Research Center of Cardiovascular Diseases, State Key Laboratory of Cardiovascular Disease, Fuwai Hospital, National Center for Cardiovascular Diseases, Chinese Academy of Medical Sciences and Peking Union Medical College, Beijing, China) for their technical assistance with data cleaning and statistical support. We acknowledge all participating hospitals and patients for their contributions to the trial. We appreciate Dr Hao Tang and Dr Yizhe Zhou’s contribution to the development of the meta-analyses.

[4242 words (of max 5000, including main text, references and figure legends)]

# ABSTRACT

**BACKGROUND** The RIGHT trial was designed to assess the efficacy and safety of postprocedural anticoagulation (PPA) in patients with ST-segment elevation myocardial infarction (STEMI) undergoing primary percutaneous coronary intervention (PCI).

**OBJECTIVES** Here we report the prespecified 1-year outcomes.

**METHODS** RIGHT is an investigator-initiated, multicenter, randomized, double-blind, placebo-controlled, superiority trial conducted in 53 sites across China. STEMI patients were randomly assigned (1:1) after primary PCI to receive low-dose PPA (enoxaparin, unfractionated heparin or bivalirudin) or matching placebo for at least 48 hours. Major adverse cardiac events (MACEs) including all-cause death, non-fatal myocardial infarction, non-fatal stroke, stent thrombosis (definite), as well as urgent revascularization (any vessel), were assessed during 1-year follow-up.

**RESULTS** Over a median follow-up of 1.0 (1.0 to 1.0) year, MACE data were available for 99.2% of participants. MACEs occurred in 4.2% (63/1494) of the PPA group and 4.9% (73/1495) of the placebo group (HR, 0.86; 95% CI, 0.61 to 1.21), with no between-group difference in major bleeding (1.3% vs. 1.5%; HR, 0.87; 95% CI, 0.47 to 1.62). In the group of enoxaparin vs. placebo, we observed reduction of MACE with enoxaparin (HR, 0.53; 95% CI, 0.30 to 0.97) with no excess bleeding. Meta-analyses also showed an advantage of enoxaparin over no anticoagulation in reducing MACE at 30 days (RR, 0.635; 95% CI, 0.399 to 0.997).

**CONCLUSIONS** Low-dose PPA after primary PCI was safe but did not reduce ischemic events at 1-year follow-up. If clinically indicated, our results suggest that enoxaparin may be beneficial and warrants confirmation in future studies. (ClinicalTrials.gov, NCT03664180)

**KEY WORDS** ST-segment elevation myocardial infarction, primary percutaneous coronary intervention, postprocedural anticoagulation, follow-up

# ABBREVIATIONS AND ACRONYMS

BARC = Bleeding Academic Research Consortium

CI = confidence interval

MACE = major adverse cardiovascular events

PCI = percutaneous coronary intervention

PPA = postprocedural anticoagulation

RIGHT = Randomized comparison of anticoagulation after primary percutaneous coronary Intervention using enoxaparin, ACT Guided unfractionated Heparin or bivalirudin prolongation vs. no anticoagulation To improve clinical outcome

STEMI = ST-segment elevation myocardial infarction

UFH = unfractionated heparin

# INTRODUCTION

Empirical prescription of postprocedural anticoagulation (PPA) after primary percutaneous coronary intervention (PCI) is frequent, aiming at preventing persistent thrombin generation after ST-segment elevation myocardial infarction (STEMI).^1^ However, data regarding the efficacy, safety and optimal regimens of routine PPA are scarce, especially when it comes to long-term follow-up. The current European and U.S. guidelines do not provide recommendations for PPA in STEMI patients.^2,3^

Results of post-hoc studies in a large patient population undergoing primary PCI by means of full-dose or low-dose post-procedural bivalirudin in comparison with unfractionated heparin (UFH) have been cited by the clinical and scientific communities to provide objective evidence in STEMI. Thus, we performed a multicenter, randomized RIGHT trial (Comparison of Anticoagulation Prolongation vs no Anticoagulation in STEMI Patients After Primary PCI) to compare PPA and placebo after a full-dose bivalirudin infusion following primary PCI. Overall, 30-day results showed no substantial clinical differences between routine PPA and placebo in this low-to-moderate risk population, although heterogeneity was observed across the three anticoagulants used.^4^

In fact, only six randomized trials administering PPA after primary PCI have been conducted, with different anticoagulants and discordant results.^4-11^ We undertook the RIGHT trial to compare the efficacy and safety of PPA and each anticoagulant in STEMI patients. We hypothesized that PPA with specific anticoagulants was superior to no anticoagulation in reducing the incidence of major adverse cardiovascular events.

# METHODS

## Study design and patients

The RIGHT trial was an investigator-initiated, multicenter, randomized, double-blind, placebo-controlled, superiority trial in STEMI patients performed in 53 centers across China. The study design, eligibility criteria, endpoints, and statistical analysis were described previously, and patient outcomes to 30 days have been reported.^4,12^ Patients eligible for randomization were those who had STEMI and underwent primary PCI within 12 hours, excluding those in shock and those who received lytic therapy prior to catheterization. Enrolled patients were randomized at 1:1 to receive low-dose PPA or matching placebo for at least 48 hours. Prior to trial initiation, each center selected one from the following three PPA regimens: enoxaparin 40 mg once daily subcutaneously, unfractionated heparin 10 units/kg/hour intravenously adjusted to maintain activated clotting time between 150 and 220 seconds, and bivalirudin 0.2 mg/kg/hour intravenously. Study assessments were performed at baseline, at 48 hours or discharge (whichever was earlier) and at 30-day, 6-month, and 1-year follow-up.

All patients or their legal representatives provided written informed consent. All participating centers obtained ethics approval from institutional review boards. Study oversight consisted of a steering committee that provided scientific direction, a clinical events committee that adjudicated selected safety and efficacy endpoints while blinded to the assigned study drug, and an independent data safety monitoring board that reviewed the endpoints and serious adverse events throughout the trial. The RIGHT trial was sponsored by Beijing Hospitals Authority Clinical Medicine Development of special funding support and Jiangsu Hengrui Pharmaceuticals provided funding for the trial through a research grant to the Beijing United Heart Foundation. The ACTION study group provided methodological, statistical, operational support for the conduct of the trial as well as editorial assistance for the manuscript. The RIGHT trial was registered at ClinicalTrials.gov (NCT 03664180) and has been completed.

## Study endpoints

The primary outcomes were collected at the 30-day follow-up, including the efficacy endpoint of major adverse cardiac events (MACE), and the safety endpoint of BARC type 3 to 5 major bleeding.^13^ MACE was defined as a composite of all-cause death, non-fatal myocardial infarction, non-fatal stroke, stent thrombosis (definite) or urgent revascularization (any vessel). All primary outcomes, as well as individual events, were re-evaluated at 1-year follow-up with the same procedure and checked by the Chinese Center for Disease Control and Prevention's national death surveillance system.^14^ This study presents the prespecified final 1-year outcomes of the entire project.

## Statistical analysis

Primary analyses of the efficacy and safety endpoints were performed in the intention-to-treat and safety populations, respectively. The cumulative incidences of the primary efficacy and safety endpoints were estimated using the Kaplan–Meier method and compared with the log-rank test. The hazard ratios and 95% confidence intervals (CI) were estimated using Cox proportional-hazards models, with adjustments made for study center as a random effect. The hazard ratio and 95% CI for the secondary ischemic and safety endpoints were estimated using mixed effects Cox proportional hazard regression models as described above. Hartung-Knapp-Sidik-Jonkman estimator was used to estimate between-study variance in our meta-analysis. The proportional hazards assumption for the Cox models was assessed using the Schoenfeld residual test, and no violation was found (Supplemental table 1). We assessed the secondary endpoints according to a hierarchical procedure to control for multiple comparisons. In this procedure, P value was reported only until the last comparison for which the P value was significant. Further details can be found in the Statistical Analysis Plan.^4^ Missing data at baseline were not imputed. Missing data for the primary endpoint were censored at the time of the last available information.

Sensitivity analyses were conducted for all study endpoints in the per-protocol population. In addition, sensitivity analyses on the primary efficacy and safety endpoints were performed using Cox models adjusted for age, gender, body mass index, diabetes, hypertension, peripheral artery disease, previous stroke, and study center as a random effect. We also performed stratified analyses in the following prespecified subgroups using multiplicative interactions terms: age (>75 vs. ≤75 years), sex, history of diabetes mellitus (yes vs. no), prior cancer (yes vs. no), prior stroke (yes vs. no), prior PCI (yes vs. no), body weight (<60 kg vs. ≥60 kg), creatinine clearance (<30 mL/min vs. ≥30 mL/min), location of myocardial infarction (anterior vs. non-anterior), anticoagulant before angiography (yes vs. no), glycoprotein IIb/IIIa inhibitor bailout use (yes vs. no), type of stent (drug-eluting stent vs. bare-metal stent), and total length of stent (>60 mm vs. ≤60 mm). All analyses were 2-sided at a 5% significance level and performed using SAS version 9.4.

## Meta-analyses

Additionally, we conducted a quantitative meta-analysis by combining our results with those of previous STEMI trials performed in patients undergoing primary PCI with PPA by calculating the risk ratio (RR) and 95% CIs for each trial (Supplemental Table 2). The PubMed, MEDLINE and EMBASE international databases were systematically searched for STEMI studies that reported PPA from their inception until June 2024. Two authors (Y.Z. and H.T.) independently conducted the search at title and abstract levels. Studies potentially eligible were independently reviewed by these authors at the full-text level and subsequently included. Any disagreements were resolved by a third senior author (Y.Y.). The primary inclusion criterion was the availability of sufficient raw data to calculate study-specific risk ratios for 30-day MACE (Supplemental Methods). Only studies involving patients undergoing primary PCI were included. The quality of the included studies was independently assessed by two reviewers using the Cochrane Risk of Bias (RoB) tool.^15^ Two authors independently extracted data from each study by reviewing the published papers and supplementary materials (Supplemental Methods, Supplemental Figure 1). Statistical heterogeneity was summarized using the I^2^ statistic test. An I^2^ value <25% was considered to indicate low heterogeneity, a value between 25% and 50% was considered moderate heterogeneity, and a value >50% was considered substantial heterogeneity, according to the Cochrane guidelines. If between-study heterogeneity was not substantial, the fixed-effect model with the Mantel-Haenszel method was used to calculate the summarized RR; otherwise, the random effects model was used. Inverse-variance weighting was used in the meta-analysis. Publication bias was evaluated by a funnel plot, Begg’s test and Egger’s test. To further compare the effectiveness of each specific regimen with that of placebo, a network meta-analysis was conducted. First, network geometry was used to explore the comparative relationships among interventions. Then, a Bayesian network meta-analysis model was used to synthesize the study effect sizes. RRs with 95% CIs for MACE were presented for each intervention comparison. Consistency evaluation was conducted by node-splitting analysis. Finally, intervention ranking was assessed using the surface under the cumulative ranking curve (SUCRA) plot, where values closer to 1 indicate superior efficacy. This systematic review and network meta-analysis followed the Preferred Reporting Items for Systematic Reviews and Meta-Analyses (PRISMA) guidelines for network meta-analyses.^16^ The P values were 2-tailed, reaching a statistically significant level at 0.05. The meta-analysis and Bayesian network meta-analyses were conducted using R 4.4.1 (R Foundation for Statistical Computing, Vienna, Austria).

# RESULTS

## Baseline characteristics

Between January 11, 2019, and September 18, 2021, 2989 participants were enrolled and assigned to either the PPA group (n=1494) or the placebo group (n=1495). Baseline demographics and procedural characteristics were similar between the two groups (Supplemental Table 3). The median duration of the study medication administration was consistent across both the PPA and the placebo groups (48.0 [48.0, 56.2] and 48.0 [48.0, 57.1] hours, respectively) and was similar among the different anticoagulants (enoxaparin, UFH or bivalirudin; 48.1 [48.0, 72.0], 48.0 [48.0, 48.9], or 48.0 [48.0, 50.0] hours, respectively). Data for the primary outcome were available for 2964 (99.2%) of the 2989 participants at the 1-year follow-up (completed in November, 2022), compared with 2980 (99.7%) of the 2989 participants at the 30-day follow-up (Supplemental Figure 2).

## 1-year Clinical outcomes

Over a median follow-up of 1.0 (1.0 to 1.0) year, MACE occurred in 4.2% (63/1494) of the patients treated with PPA compared with 4.9% (73/1495) of the patients treated with placebo (hazard ratio, 0.86; 95% CI, 0.61 to 1.21) (Table 1 and Fig. 1A). The incidence of BARC 3 to 5 bleeding did not differ significantly between patients who received PPA and those who did not (1.3% [19/1468] vs. 1.5% [22/1488]; hazard ratio, 0.87; 95% CI, 0.47 to 1.62) (Table 1 and Fig. 1B). The event rates of the other endpoints are shown in Table 1.

As shown in Supplemental Figure 3A and 3B, the effects of PPA versus placebo on the efficacy and safety endpoints were largely consistent across the prespecified subgroups.

There was apparent heterogeneity in the effect of the three anticoagulants over 1-year follow-up (Fig. 2 and Supplemental Table 4). The incidence of the primary efficacy endpoint was lower with enoxaparin than placebo (3.6% [17/474] vs. 6.6% [31/471]; hazard ratio, 0.53; 95% CI, 0.30 to 0.97), while no significant difference was observed with bivalirudin vs. placebo (hazard ratio, 1.12; 95% CI, 0.66 to 1.88) and UFH vs. placebo (hazard ratio, 1.08; 95% CI, 0.53 to 2.18) (Fig. 3).

## Meta-analysis

A total of 791 studies were initially identified through comprehensive searches across multiple databases. After removing duplicated studies, 573 studies remained. Following title and abstract screening, 162 articles were excluded. After full-text assessment, an additional 155 studies were removed based on the eligibility criteria. A total of 7 studies were included in the analysis (Supplemental Figure 4). Differences in baseline characteristics, designs, and results are shown in Supplemental Table 2. Funnel plot, Begg’s test (P=0.55) and Egger’s test (P=0.87) indicated there was no significant publication bias among all included studies (Supplemental Figure 5). The I^2^ was 50.6% (0.0%-79.0%) and τ² was 0.034 (0.000-0.256), which indicated significant between-study heterogeneity.

The overall risk of MACE at 30 days was not significantly different between patients with PPA and those without (RR, 1.05; 95% CI 0.89 to 1.25), as shown in Fig. 4A. There was significant heterogeneity across the three anticoagulants in terms of MACE at 30 days (Fig. 3; Fig. 4B, C, and D). No significant inconsistency was identified based on node-splitting analysis (P=0.90). Postprocedural enoxaparin was associated with a lower risk (RR, 0.635; 95% CI, 0.399 to 0.997), while UFH and bivalirudin exhibited RRs of 0.969 (95% CI, 0.592 to 1.53) and 0.988 (95% CI, 0.851 to 1.15), respectively.

# DISCUSSION

We conducted a randomized trial comparing low-dose PPA (using three different anticoagulants) with no anticoagulation (matching placebos) in low-to-moderate risk patients with STEMI (Central Illustration). Although low-dose PPA administered for at least 48 hours after primary PCI was safe, it did not prove superior to no anticoagulation in reducing ischemic outcomes at 1 year. However, low-dose enoxaparin improved the risk-benefit ratio compared to placebo at 1 year in contrast to the other two anticoagulants. Our results in the RIGHT trial seem to be confirmed by the meta-analyses of six randomized trials.

Our 1-year analysis, with no time-dependent divergence, confirms the 30-day findings, indicating that low-dose PPA provides no benefit in reducing ischemic events and shows no sign of harm in patients with STEMI. In contrast to previous studies, participants in our trial were eligible for randomization only if they received intravenous bivalirudin during and immediately after primary PCI, with a full dose of 1.75 mg/h required prior to PPA initiation. ^8-11,17^ Despite consistent enrollment criteria with previous trials, the baseline characteristics may reflect a selection bias, as centers appeared to recruit patients at relatively low risk following successful procedures, potentially attenuating the benefit of PPA. In high-risk STEMI presentations (such as cardiogenic shock, angiographic no-reflow, or left ventricular thrombus) PPA has been reported in selected cohorts and may be associated with potential clinical benefit.^18,19^ Future studies with comprehensive procedural and angiographic data collection will be required to definitively evaluate treatment effects within the complex high-risk PCI population.

Extended follow-up up to 1 year confirmed the findings observed at 30 days, showing that PPA was not superior to placebo. However, the three anticoagulants behaved differently in preventing ischemic events over 1 year follow-up, confirming the 30-day data. Enoxaparin demonstrated a lower incidence of MACEs at 30 days, with a consistent reduction at 1 year. These findings are consistent with those of previous studies, but should be interpreted with caution. Although age and prior MI were assessed for transitivity, key effect modifiers—such as No. of premature termination of investigational products, duration of investigational products—were poorly reported in most studies, which limited the reliability of indirect comparisons (Supplemental Table 4). Although pre-specified the comparison between anticoagulants remains hypothesis generating, given the study design and the lack of power for interaction analysis.^20-22^ Nevertheless, in centers allocated to enoxaparin, the randomized double-blind comparison favored enoxaparin PPA against placebo, a result not observed in centers allocated to UFH or bivalirudin PPA.

A biologically plausible explanation for the observed separation with enoxaparin is that, in the hours-to-days after PCI—when tissue-factor–driven coagulation remains heightened and dual antiplatelet therapy primarily targets platelet activation—low–molecular-weight heparin may provide a steadier “bridging” inhibition of coagulation through its more predictable anti–coagulation compared to UFH and broader inhibition (anti-factor Xa and anti-IIa) compared to bivalirudin. In addition, this is obtained without infusion and with lower interpatient variability (less nonspecific protein/cell binding) and without anticoagulation monitoring). These results are aligned with similar superiority of enoxaparin reported in the past.^6,9-11,22^ Given the significance of this question, we updated the previous literature search and network meta-analysis to provide a state-of-the-art evidence base on different types of PPA in the STEMI population. In this updated network meta-analysis, a parenteral anticoagulant regimen that includes prophylactic use of enoxaparin after primary PCI appears to be associated with fewer ischemic complications while maintaining safety. Although, heterogeneity sources given substantial clinical differences across trials (e.g., GPI use 3.3-77%, radial access 23.8-98.6%), our data suggest that a regimen of 40 mg enoxaparin o.d. has the best safety profile (NNT=33.3 for MACE) and may represent a promising strategy that warrants evaluation in adequately powered, confirmatory randomized trials in the future.

Most guideline recommendations for PPA are based on low-quality evidence, often relying on extrapolation from observational studies. Remarkable advances have been made in recent years, starting with the initial findings from the MATRIX trial in patients with acute coronary syndrome, followed by the RIGHT trial in STEMI.^4,17^ Together, these studies provide high-quality data from more than 21,000 patients, offering meaningful insights. Given these findings and in line with the latest European Society of Cardiology guidelines, PPA is broadly not recommended for routine use after invasive procedures, especially in patients at low risk or recurrent events.^2^

## Study limitations

The design of the RIGHT trial resulted in a selective population with a low event rate, which may limit the generalizability of the findings to broader populations. The trial population was restricted to patients enrolled in China; therefore, the generalizability of these findings to other geographic regions and ethnic populations remains uncertain. Additionally, the three anticoagulants were not directly comparable due to the absence of central randomization between the three anticoagulants (for practical reasons) but all patients were randomized between one anticoagulant and the matching placebo, and the trial was not powered for interaction analysis. The double-blind nature of the study the blinded event adjudication by the clinical event committee ensures however the absence of bias in the reported events. Considering the inherent difficulties of a central randomization for the type of drug (emergent situation, blinded pharmaceutical circuit for three drugs and three placebos at each center, cost issues etc…) the type of anticoagulant was not centrally allocated, but local randomization and blinding occurred for each patient for the drug selected by the center. We cannot exclude inherent center-level confounding with this design. While the use of network meta-analysis enables simultaneous comparisons and evidence-based grading to facilitate overall conclusions, we believe it lacks the granularity to address specific anticoagulants. Another limitation is the lower-than-expected event rate, as frequently encountered in recent acute MI trials. This may have reduced statistical power and contributed to the wide confidence interval around the hazard ratio. Based on our observed effect estimate (HR 0.53) and 1-year MACE rates (3.6% with enoxaparin vs 6.6% with placebo), a confirmatory trial would require approximately 859 patients per group (≈1,718 total) to ensure adequate power.

# CONCLUSION

Our results complement previous work suggesting that routine PPA after primary PCI is safe but does not improve ischemic outcomes. The data are consistent throughout the trial from 30 days to 1 year, and in the network meta-analysis. Moreover, in cases where PPA is clinically warranted, our results consistently indicate that enoxaparin effectively reduces the risk of MACE after primary PCI.

# **REFERENCES**

1. Yan Y, Gong W, Ma C, et al. Postprocedure Anticoagulation in Patients With Acute ST-Segment Elevation Myocardial Infarction Undergoing Primary Percutaneous Coronary Intervention. JACC Cardiovascular Interventions 2022;15:251-263.

2. Byrne RA, Rossello X, Coughlan JJ, et al. 2023 ESC Guidelines for the management of acute coronary syndromes. Eur Heart J 2023;44:3720-3826.

3. Levine GN, Bates ER, Blankenship JC, et al. 2015 ACC/AHA/SCAI Focused Update on Primary Percutaneous Coronary Intervention for Patients With ST-Elevation Myocardial Infarction: An Update of the 2011 ACCF/AHA/SCAI Guideline for Percutaneous Coronary Intervention and the 2013 ACCF/AHA Guideline for the Management of ST-Elevation Myocardial Infarction. J Am Coll Cardiol. 2016 Mar 15;67(10):1235-1250.

4. Yan Y, Guo J, Wang X, et al. Postprocedural Anticoagulation After Primary Percutaneous Coronary Intervention for ST-Segment-Elevation Myocardial Infarction: A Multicenter, Randomized, Double-Blind Trial. Circulation 2024;149:1258-1267.

5. Batchelor WB, Mahaffey KW, Berger PB, et al. A randomized, placebo-controlled trial of enoxaparin after high-risk coronary stenting: the ATLAST trial. J Am Coll Cardiol 2001;38:1608-13.

6. Montalescot G, Zeymer U, Silvain J, et al. Intravenous enoxaparin or unfractionated heparin in primary percutaneous coronary intervention for ST-elevation myocardial infarction: the international randomised open-label ATOLL trial. Lancet 2011;378:693-703.

7. Li Y, Liang Z, Qin L, et al. Bivalirudin plus a high-dose infusion versus heparin monotherapy in patients with ST-segment elevation myocardial infarction undergoing primary percutaneous coronary intervention: a randomised trial. Lancet 2022;400:1847-1857.

8. Chang CC, Chichareon P, Modolo R, et al. Association between post-percutaneous coronary intervention bivalirudin infusion and net adverse clinical events: a post hoc analysis of the GLOBAL LEADERS study. Eur Heart J Cardiovasc Pharmacother 2020;6:22-30.

9. Steg PG, van 't Hof A, Hamm CW, et al. Bivalirudin started during emergency transport for primary PCI. N Engl J Med 2013;369:2207-2217.

10. Valgimigli M, Frigoli E, Leonardi S, et al. Bivalirudin or Unfractionated Heparin in Acute Coronary Syndromes. N Engl J Med 2015;373:997-1009.

11. Ducrocq G, Steg PG, Van't Hof A, et al. Utility of post-procedural anticoagulation after primary PCI for STEMI: insights from a pooled analysis of the HORIZONS-AMI and EUROMAX trials. Eur Heart J Acute Cardiovasc Care 2017;6:659-665.

12. Yan Y, Wang X, Guo J, et al. Rationale and design of the RIGHT trial: A multicenter, randomized, double-blind, placebo-controlled trial of anticoagulation prolongation versus no anticoagulation after primary percutaneous coronary intervention for ST-segment elevation myocardial infarction. Am Heart J 2020;227:19-30.

13. Mehran R, Rao SV, Bhatt DL, et al. Standardized bleeding definitions for cardiovascular clinical trials: a consensus report from the Bleeding Academic Research Consortium. Circulation 2011;123:2736-47.

14. Zhou M, Wang H, Zeng X, et al. Mortality, morbidity, and risk factors in China and its provinces, 1990-2017: a systematic analysis for the Global Burden of Disease Study 2017. Lancet 2019;394:1145-1158.

15. Sterne JAC, Savović J, Page MJ, et al. RoB 2: a revised tool for assessing risk of bias in randomised trials. BMJ. 2019;366:l4898.

16. Hutton B, Salanti G, Caldwell DM, et al. The PRISMA extension statement for reporting of systematic reviews incorporating network meta-analyses of health care interventions: checklist and explanations. Ann Intern Med. 2015;162(11):777-84.

17. Gargiulo G, Carrara G, Frigoli E, et al. Post-Procedural Bivalirudin Infusion at Full or Low Regimen in Patients With Acute Coronary Syndrome. J Am Coll Cardiol 2019;73:758-774.

18. Zhang Z, Si D, Zhang Q, et al. Prophylactic Rivaroxaban Therapy for Left Ventricular Thrombus After Anterior ST-Segment Elevation Myocardial Infarction. JACC Cardiovasc Interv. 2022;15(8):861-872.

19. Gong W, Yan Y, Wang X, et al. Risk Factors for In-Hospital Cardiac Arrest in Patients With ST-Segment Elevation Myocardial Infarction. J Am Coll Cardiol. 2022;80(19):1788-1798.

20. Collet J-P, Huber K, Cohen M, et al. A direct comparison of intravenous enoxaparin with unfractionated heparin in primary percutaneous coronary intervention (from the ATOLL trial). Am J Cardiol 2013;112:1367-1372.

21. Montalescot G, Ellis SG, de Belder MA, et al. Enoxaparin in primary and facilitated percutaneous coronary intervention A formal prospective nonrandomized substudy of the FINESSE trial (Facilitated INtervention with Enhanced Reperfusion Speed to Stop Events). JACC Cardiovascular Interventions 2010;3:203-212.

22. Silvain J, Beygui F, Barthélémy O, et al. Efficacy and safety of enoxaparin versus unfractionated heparin during percutaneous coronary intervention: systematic review and meta-analysis. BMJ 2012;344:e553.

# FIGURE TITLES AND CAPTIONS

**Figure 1.** **1-Year Clinical Outcomes in** **Postprocedural anticoagulation versus Placebo Group**

Primary objective, Postprocedural anticoagulation (PPA) versus placebo: MACE: all-cause death, non-fatal myocardial infarction, non-fatal stroke, stent thrombosis (definite) or urgent revascularization (any vessel) over 1-year of follow-up (Panel A); BARC 3 to 5 bleeding through 1 year from randomization (Panel B).  BARC denotes Bleeding Academic Research Consortium, CI confidence interval, and PPA postprocedural anticoagulation.

**Figure 2. 1-Year Clinical Outcomes in Three Anticoagulation Regimens**

MACE: all-cause death, non-fatal myocardial infarction, non-fatal stroke, stent thrombosis (definite) or urgent revascularization (any vessel) over 1-year of follow-up (Panel A); BARC 3 to 5 bleeding through 1 year from randomization (Panel B).

**Figure 3. Efficacy and safety outcomes in three anticoagulation regimens versus placebo**

Key secondary objective, effect of each anticoagulation regimen (enoxaparin, UFH, or bivalirudin) versus placebo: MACE of all-cause death, non-fatal myocardial infarction, non-fatal stroke, stent thrombosis (definite) or urgent revascularization (any vessel) over 1-year of follow-up (Panel A); BARC 3 to 5 bleeding through 1 year from randomization (Panel B).

**Figure 4.** **Meta-analyses**

Meta-analysis estimates of PPA effectiveness in MACE during the 30-day period compared with no PPA (A) or network analysis of three anticoagulation treatments (enoxaparin, UFH, or bivalirudin) versus placebo with network plot (B), ranking plot (C), and forest plot (D). A regimen that includes enoxaparin seems to be the most favorable treatment option and may be the preferred PPA for most of STEMI patients after primary PCI.

**Central Illustration. Key Findings**

Study design and 1-year outcomes of the RIGHT trial. A total of 2,989 patients with ST-segment elevation myocardial infarction (STEMI) undergoing primary percutaneous coronary intervention (PCI) with bivalirudin were randomized to placebo or low-dose postprocedural anticoagulation with unfractionated heparin (UFH), enoxaparin, or bivalirudin, according to center assignment. Kaplan-Meier curves show no significant difference between groups in major adverse cardiovascular events (MACE) or Bleeding Academic Research Consortium (BARC) type 3 to 5 bleeding at 1 year. The Meta-analyses from prior randomized trials suggests a reduction in 30-day MACE with enoxaparin versus no anticoagulation.

**Table 1. Clinical Outcomes in the Global Population at 1 Year***

| **Outcomes** | **PPA** | | **Placebo** | | **Hazard ratio**  **(95% CI)** | ***P* Value** |
| --- | --- | --- | --- | --- | --- | --- |
|  | **No. of patients with event** | **Outcome rates and 95% CI derived from Kaplan-Meier analysis** | **No. of patients with event** | **Outcome rates and 95% CI derived from Kaplan-Meier analysis** |  |  |
| Efficacy endpoints | n=1494 |  | n=1495 |  |  |  |
| MACE | 63 | 4.2% (3.1% - 5.2%) | 73 | 4.9% (3.6% - 5.8%) | 0.86 (0.61, 1.21) | 0.383 |
| All-cause death, non-fatal MI, non-fatal stroke, or uR | 63 | 4.2% (3.1% - 5.2%) | 73 | 4.9% (3.6% - 5.8%) | 0.86 (0.61, 1.21) |  |
| All-cause death, non-fatal MI, or non-fatal stroke | 61 | 4.1% (3.1% - 5.2%) | 72 | 4.8% (3.5% - 5.7%) | 0.84 (0.6, 1.19) |  |
| Cardiovascular death or non-fatal MI | 48 | 3.2% (2.0% - 3.8%) | 44 | 2.9% (2.4% - 4.3%) | 1.21 (0.76, 1.90) |  |
| Definite stent thrombosis (ARC definition) | 4 | 0.3% (0.0% - 0.5%) | 4 | 0.3% (0.0% - 0.6%) | 1.00 (0.25, 4.00) |  |
| Cardiovascular death | 41 | 2.7% (2.0% - 3.8%) | 34 | 2.3% (1.4% - 2.9%) | 1.21 (0.77, 1.90) |  |
| All-cause death | 43 | 2.9% (2.1% - 3.9%) | 37 | 2.5% (1.5% - 3.1%) | 1.16 (0.75, 1.81) |  |
| Non-fatal MI | 17 | 1.1% (0.5% - 1.6%) | 26 | 1.7% (1.1% - 2.5%) | 0.65 (0.35, 1.20) |  |
| Non-fatal stroke | 10 | 0.7% (0.2% - 1.0%) | 11 | 0.7% (0.2% - 1.1%) | 0.91 (0.39, 2.14) |  |
| Safety endpoints | n=1468 |  | n=1488 |  |  |  |
| BARC 3 to 5 bleeding | 19 | 1.3% (0.7% - 1.9%) | 22 | 1.5% (0.9% - 2.1%) | 0.87 (0.47, 1.62) | 0.67 |
| BARC 1, 2, 3, 4, and 5 | 215 | 14.6% (13.0% - 16.6%) | 217 | 14.6% (12.9% - 16.6%) | 1.01 (0.84, 1.22) |  |
| BARC 2, 3 or 5 | 59 | 4.0% (3.0% - 5.1%) | 58 | 3.9% (2.9% - 4.9%) | 1.04 (0.72, 1.49) |  |
| TIMI major, minor, minimal bleeding and their combination | 60 | 4.1% (3.1% - 5.1%) | 58 | 3.9% (2.9% - 4.9%) | 1.05 (0.73, 1.51) |  |
| STEEPLE major bleeding | 18 | 1.2% (0.7% - 1.8%) | 22 | 1.5% (0.9% - 2.1%) | 0.83 (0.44, 1.54) |  |
| GUSTO severe or moderate bleeding | 12 | 0.8% (0.4% - 1.3%) | 12 | 0.8% (0.4% - 1.3%) | 1.01 (0.45, 2.25) |  |
| Thrombocytopenia | 4 | 0.3% (0.0% - 0.5%) | 0 | 0.0% (0.0% - 0.0%) | - |  |

ARC indicates Academic Research Consortium; BARC, Bleeding Academic Research Consortium; GUSTO, Global Utilization of Streptokinase and Tissue Plasminogen Activator for Occluded Coronary Arteries; MACE, all-cause death, non-fatal myocardial infarction, non-fatal stroke, stent thrombosis (definite) or urgent revascularization (any vessel);MI, myocardial infarction; uR, urgent revascularization; PPA, post-procedural anticoagulation; and STEEPLE, Safety and Efficacy Of Enoxaparin In Percutaneous Coronary Intervention Patients and TIMI Thrombolysis in Myocardial Infarction.

* The analysis of the efficacy indicators was carried out in ITT set and analysis of the safety endpoints were carried out in the safety set.
